# Supplementary material for: A High-Throughput Yellow Fever Neutralization Assay
Source: Microbiol Spectr. 2022 Jun 7;10(3):e02548-21. doi: 10.1128/spectrum.02548-21 (PMC9241659; doi:10.1128/spectrum.02548-21)
Supplement: Supplemental file 1 — Supplemental material. Download spectrum.02548-21-s0001.pdf, PDF file, 1.1 MB [file spectrum.02548-21-s0001.pdf]

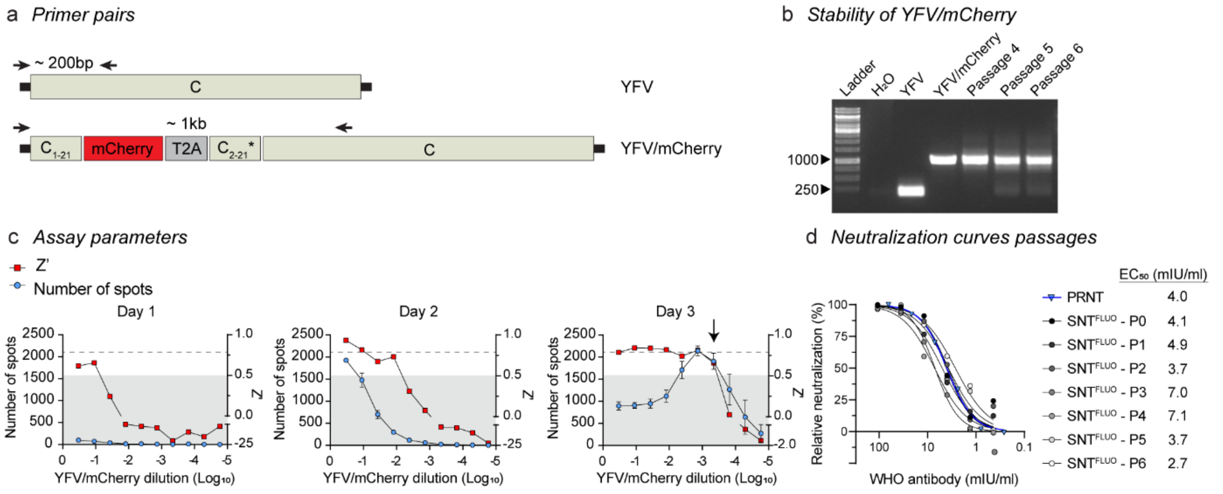

Figure S1. Optimization of SNT^{FLUO}. a, Schematic representation of RT-PCR based detection of the mCherry insert. Arrows indicate primer binding sites on the viral genome. **b,** RT-PCR fingerprint of viral RNA extracted from infected BHK-21J supernatants of serially passaged YFV/mCherry (P4–P6). PCR amplicons of pShuttle-YFV and pShuttle-YFV/mCherry were amplified using the same primer pair and served as positive controls. H_2O was included as a negative control. ladder, 1-kb DNA ladder. Data are from a single representative experiment. **c,** BHK-21J cells were infected with serially diluted YFV/mCherry. Spot counts (left Y-axis) and Z' (right Y-axis) at indicated time points post infection. Grey area indicates Z' values <0.5. Dashed line indicates saturation point of number of spots (~2000 spots/well of a microtiter plate). Arrow in right panel indicates the highest Z' value at the lowest virus dilution, immediately below a saturation point and was chosen for further assay development and validation, corresponding to a MOI of 0.02 as measured with classical plaque assay. Data are the means ± standard deviations of three replicates.

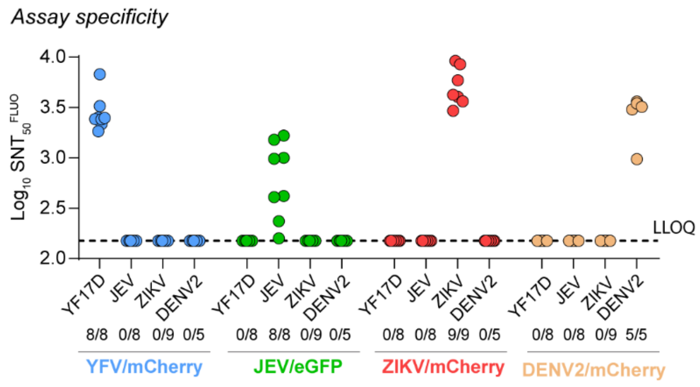

Figure S2. Specificity of SNT^{FLUO} against other flaviviruses. Scatter plot of individual neutralizing titers (SNT_{50}^{FLUO}) for 30 serum samples from mice vaccinated with either YF17D (n=8), JEV (n=8), ZIKV (n=9) and DENV2 (n=5), and tested against YFV/mCherry (MOI 0.02), JEV/eGFP (MOI 0.04), ZIKV/mCherry (determination of MOI not possible) and DENV2/mCherry (MOI 4.5). The number of seroconverted animals is shown as well. Data are the means of three replicates. LLOQ - Lower limits of quantification (dashed black line).

Table S1. SNT^{FLUO} validation by reference laboratories. Neutralizing titers of 1:10 serially diluted sera (1:10^1 – 1:10^4) from four non-vaccinated and six YF17D-vaccinated NHP. Data are the means of six (ITM and our laboratory) or two (Sciensano) replicates.

**Monkey ID**	**Day post-vaccination**	**log_{10} NT50-FLUO**	**log_{10} $PRNT_{50}$**	**log_{10} $RFFIT_{50}$**
R08086	42	4,2	>3,1	>2,9
R08086 1:10	42	*3,0**	*3,0**	*2,2**
R08086 1:100	42	*1,6**	*1,9**	<1,8
R08086 1:1000	42	<1	1,7	<1,8
R08086 1:10000	42	<1	<1,6	<1,8
R09108	42	>4,6	>3,1	>2,9
R09108 1:10	42	*3,3**	*3,0**	*2,7**
R09108 1:100	42	*2,0**	*2,1**	*1,8**
R09108 1:1000	42	<1	1,8	<1,8
R09108 1:10000	42	<1	<1,6	<1,8
R09131	42	>4,6	>3,1	>2,9
R09131 1:10	42	*4,0**	*3,0**	*2,7**
R09131 1:100	42	*2,4**	*2,4**	*1,8**
R09131 1:1000	42	<1	1,8	<1,8
R09131 1:1000	42	<1	<1,6	<1,8
R04068	72	3,8	>3,1	>2,9
R04068 1:10	72	*2,8**	*3,0**	*2,4**
R04068 1:100	72	*1,6**	*2,1**	<1,8
R04068 1:1000	72	<1	1,7	<1,8
R04068 1:10000	72	<1	<1,6	<1,8
R06081	72	4,5	>3,1	>2,9
R06081 1:10	72	*3,5**	>3,1	*2,4**
R06081 1:100	72	*2,0**	*2,4**	<1,8
R06081 1:1000	72	<1	1,7	<1,8
R06081 1:10000	72	<1	<1,6	<1,8
R12037	72	4,2	>3,1	>2,9
R12037 1:10	72	*3,3**	*2,8**	*2,6**
R12037 1:100	72	*1,8**	*2,1**	<1,8
R12037 1:1000	72	<1	<1,6	<1,8
R12037 1:10000	72	<1	<1,6	<1,8
R02006	42	<1	<1,6	<1,8
R07066	72	<1	<1,6	<1,8
R11050	72	<1	<1,6	<1,8
Pooled negative serum	72	<1	<1,6	<1,8

*used for correlation analysis

Protocol Exchange

Materials

Biological materials

- BHK-21J cells (gift from Prof. Peter Bredenbeek, LUMC, NL)
- pShuttle-YFV/mCherry plasmid
- YFV/mCherry virus stock
- Serum samples after YF17D-vaccination

Reagents

- Dulbecco's Phosphate buffered saline (DPBS; Gibco, cat. no.14190094)
- Minimum Essential Medium (MEM; Gibco, cat. no.21090022)
- Fetal Bovine Serum (FBS; HyClone, SV30160.03)
- L-glutamine, 200 mM (Gibco, cat. no.25030149)
- Sodium bicarbonate, 7.5% solution (Gibco, cat. no.25080060)
- HEPES buffer solution, 1M solution (Gibco, cat. no.15630056)
- MEM non-essential amino acids solution (NEAA; Gibco, cat. no.11140035)
- Penicillin-streptomycin solution, 10,000 U/ml solution (PenStrep; Gibco, 15140148)
- Trypsin-EDTA, 0.05% solution (Gibco, cat. no.25300054)
- Formaldehyde, 37% solution (Sigma-Aldricht, cat. no.252549)
- TransIT-LT1 Transfection Reagent (Mirusbio, cat.no.MIR2305)
- Opti-MEM (Gibco, cat. no.31985062)

Consumables

- Falcon conical tubes; 15, 50 ml (Greiner Bio-One, cat. no.188271, cat. no.227261)
- 48-well PCR plate and 8-strip caps (Thermo Scientific, cat. no.AB0648, cat. no.AB0783)
- 96-well black cell culture microplates with clear bottom (Greiner Bio-One, cat. no.655090)

- 96-well clear round bottom microplates (Corning, cat. no.3799)
- Sterile filter pipettes; 10, 25, 50 ml (VWR, cat. no.7341738, cat. no.7341739, cat. no.7341740)
- Sterile pipettes without filter; 2 ml (VWR, cat. no.7340456)
- Sterile filter tips; 0,1-20, 10-100, 100-1250 µl (VWR, cat. no.7323681, cat. no.7323683, cat. no.GREI750261_3840)
- Sterile tips without filter; 200 µl (Biotix, cat. no.M02009SC)
- Sterile disposable reagent reservoirs; 25, 50 ml (VWR, cat. no.6131174, cat. no.6131183)
- T-150 cell culture flasks (TPP, cat. no.90151)
- Cell counting slides (e.g. Logos Biosystems, cat. no. LB L12001)
- Cryotubes (VWR, cat. no.4791239)
- 5 ml Eppendorf tubes (VWR, cat. no.5250794)

Software package

- Genedata screener version 17.0.4 (Genedata AG, Basel, Switzerland)

Equipment

- Pipetboy (VWR, cat. no.612-0928)
- Single channel manual micropipettes; 2-20, 20-200, 100-1000 µl (Thermo Scientific, cat. no.4700860N).
- 8- and 12-channel electronic micropipettes; 5-100 (P100), 50-1250 (P1250) µl (Eppendorf, cat. no.6132237, cat. no.6132238, cat. no.6132241, cat. no.6132242).
- CO_2 cell culture incubator (e.g. Binder, Model CB220, cat. no.3901006)
- Class II biological safety cabinet (e.g. CleanAir)
- Fluid aspiration system (e.g. BVC professional, Vacuubrand)
- Inverted phase-contrast microscope (e.g. Primovert, Zeiss)
- Automated cell counter (e.g. Luna-II, Logos Biosystems)
- Microcentrifuge (e.g. Galaxy MiniStar, VWR)
- Centrifuge (e.g. Sorvall ST 40, Thermo Scientific)

- Thermocycler with 48-well block (Biometra TRIO, Analytik-Jena)
- Immunospot reader (e.g. CTL, cat. no.S6UTM12)
- Ultra low temperature freezer -86°C (e.g. New Brunswick Scientific, Innova Model U535)
- Fluorescent microscope (e.g. ZOE fluorescent cell imager, Bio-Rad, cat.no.1450031)
- Fume hood

Reagent setup

Δ CRITICAL *L-glutamine is unstable at 4-8°C. Thaw it prior to use and store at 4-8°C for ≤5 days.*

- *Culture medium*. To 500 ml MEM, add 50 ml of FBS and 5 ml of L-glutamine, sodium bicarbonate, HEPES, NEAA and PenStrep.

  Δ CRITICAL *Store culture medium at 4-8°C for ≤5 days before use.*

- *Assay medium*. To 500 ml MEM, add 10 ml of FBS and 5 ml of L-glutamine, sodium bicarbonate, HEPES, NEAA and PenStrep.

  Δ CRITICAL *Store assay medium at 4-8°C for ≤5 days before use.*

- *Fixation solution*. To 500 ml DPBS, add 108.1 ml of 37% formaldehyde solution. Store 8% fixation solution at room-temperature (RT).

  ! CAUTION *Formaldehyde is harmful and should be preferably handled in a fume hood. Wear gloves, and avoid contact with eyes and skin.*

Procedure

---

1. **Cell culture maintenance.**

   Timing 15-30 min (once a week)

   Δ CRITICAL:

- *All the steps must be performed in a class II biosafety cabinet, using proper aseptic technique.*
- *Allow culture medium and trypsin-EDTA solution to equilibrate to RT prior to use.*

- *Aspiration is performed with 2 ml vacuum pipet and the vacuum pump is set to maximum suction power (8 LED's, 850 mbar underpressure).*
- *Cells can be kept up to 6 months in culture and thereafter should be discarded.*

1. Inspect cells in T150 flask using inverted microscope and determine confluency. Note: usually the cells are 90-100% confluent if splitting them on a weekly basis.
2. Aspirate culture medium and briefly rinse cell layer with 10 ml of DPBS to remove serum traces.
3. Add 2 ml of 0.05% trypsin-EDTA solution and tilt the flask several times to cover the entire bottom with trypsin solution.
4. Incubate 3-5 minutes at 37°C and 5% CO_2 until the cells detach from the culture flask.
5. Add 10 ml culture medium to the flask and resuspend cells by pipetting up and down. Transfer the suspension to a 15 ml conical tube.
6. Centrifuge cells during 5 min at 400 xg.
7. Remove supernatant.
8. Add 6 ml of culture medium to the 15 ml tube and resuspend cells by pipetting up and down.
9. Add 39 ml of culture medium to a new T150 culture flask and label the flask accordingly.
10. Transfer 1 ml of cell suspension to the new culture flask (1:6 splitting).
11. Rock culture flask gently to spread cells in medium and incubate at 37°C and 5% CO_2 for 4-7 days.
12. After the end of culturing period, proceed again with the steps described in section 1.

2. Production of YFV/mCherry virus stock.

Timing 5-7 days

Δ CRITICAL:

- *All the steps must be performed in a class II biosafety cabinet, using proper aseptic technique.*
- *Allow TransIT™-LT1 Transfection Reagent, Opti-MEM, culture and assay media to equilibrate to RT prior to use.*

2.1. Cell seeding.

Timing 15 min (day -1)

1. Prepare cell culture suspension as described in section 1, and measure cell density.
2. Seed 3,5x10^6 BHK-21J cells in 40 ml of culture medium in two T150 flasks.
3. Incubate cells overnight at 37°C and 5% CO_2 to adhere cells in the flasks.

2.2. Transfection.

Timing 30 min (day 0)

1. Replace culture medium with 20 ml of assay medium and place the flasks back to the incubator.

   Δ CRITICAL *Avoid pipetting directly on cell monolayer and hold pipet against the opposite wall in the culture flask while dispensing.*

2. Combine 4 ml of Opti-MEM with and without 40 µg of pShuttle-YFV/mCherry plasmid in two sterile 5 ml tubes and mix gently by pipetting up and down.
3. Add 120 µl of TransIT™-LT1 Transfection Reagent in each tube and repeat mixing.
4. Incubate at room temperature for 20 minutes.
5. Transfer "Transfection reagent – plasmid DNA complexes" drop-wise to different areas in the medium of one of the flasks with BHK-21J cells. Label the flask with virus name.
6. Transfer "Transfection reagent" without plasmid DNA to the second flask and label it as "Mock".
7. Gently rock the flasks and incubate during 16h at 37°C.

2.3. Medium replacement.

Timing 5 min (day 1)

1. Remove medium from both flasks and wash gently with 30 ml of DPBS to remove excessive plasmid.

   Δ CRITICAL *Avoid pipetting directly on cell monolayer and hold pipet against the opposite wall in the culture flask while dispensing.*

2. Add 30 ml of assay medium.
3. Incubate for 3-5 days.

2.4. Harvesting.

Timing 1h (day 3-5)

1. Monitor mCherry signal progression daily. Use mock-transfected cells to set the background.
2. Upon onset of cytopathic effect (CPE) as shown in Figure 1, transfer supernatant with YFV/mCherry into a 50 ml Falcon tube.

A B

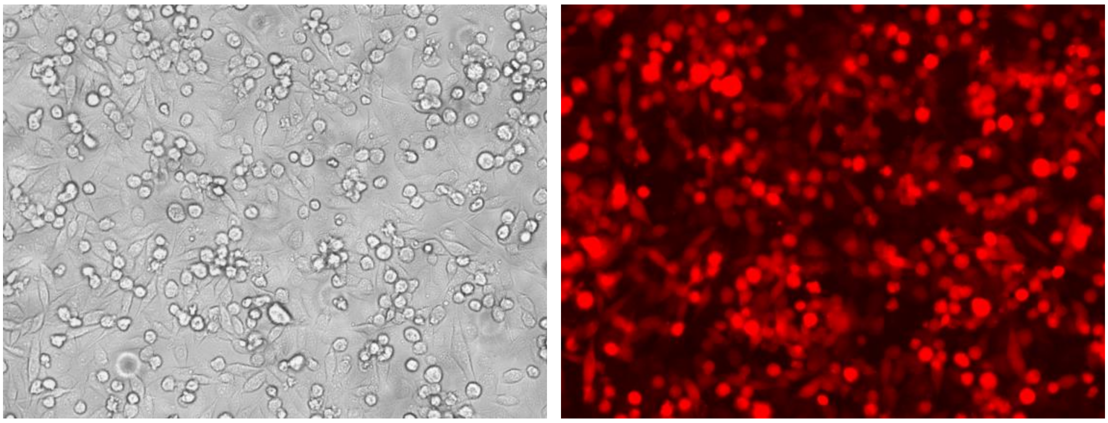

Figure 1. BHK-21J cells after 3 days post-infection with YFV/mCherry in T150 culture flask. Images were taken using ZOE cell imaging station. A – Bright field; B – Expression of mCherry linked to YFV.

3. Centrifuge 5 min at 2100g to pellet cell debris.
4. Transfer clear supernatant into a new Falcon tube, mix and aliquot in cryotubes (500µl/tube). This is passage 0 (P0).

   □ **PAUSE POINT** *Virus stock aliquots can be stored at -80°C for a long time. Once virus is thawed, do not refreeze it again.*

   Note: To produce a new batch of virus stock, either start from transfection (Section 2) or infect cells seeded as described in Section 2: 2.1 with 500 µl of P0 virus and harvest as described in Section 2: 2.4 (this will be P1). Passaging can be repeated up to P5 as the mCherry transgene remains stable at least till P6.

3. Titration of YFV/mCherry virus stock.

Timing 5 days

Δ CRITICAL:

- *All the steps must be performed in a class II biosafety cabinet, using proper aseptic technique.*
- *Allow assay medium to equilibrate to RT prior to use.*

- *Allow YFV/mCherry virus stock to thawed at RT for at least 15 min.*
- *Aspiration with 2 ml vacuum pipet is performed setting the vacuum pump to maximum suction power (8 LED's, 850 mbar underpressure).*
- *Aspiration with the 8-channel vacuum manifold is performed setting the vacuum pump to low suction power (2 LED's, 250 mbar underpressure). Hold the tips at an angle of 45 degrees and slowly move down against the well wall until the bottom is reached.*
- *Dispensing steps are performed with 8- and 12-channel electronic micropipettes (P100, P1250). Hold the tips at a 45 degree angle against the well wall during dispensing, unless otherwise mentioned.*

3.1. Cell seeding.

Timing 15 min (day -1)

1. Prepare cell culture suspension as described in section 1, and determine cell density.
2. Prepare working solution by diluting cell suspension in assay medium to reach cell density of $1x10^5$ cells/ml. Note: 10 ml of working solution is required per plate.
3. Invert the tube with diluted cells to mix and decant working solution into a reagent reservoir.
4. Add 100 µl of working solution to each well in a black 96-well microplate with clear bottom ($1x10^4$ cells/well). This is a **cell plate**.
5. Label the cell plate with cell line name and date.
6. Incubate the cell plate overnight at 37°C and 5% CO_2 to adhere cells to the bottom of wells.

3.2. Virus titration.

Timing 1h 20 min (day 0)

A) Medium replacement.

Timing 5 min (day 0)

1. Aspirate medium from cell plate.
2. Gently add 100 µl of assay medium to the cells in the marginal wells.

   Δ CRITICAL *Set the dispensing speed at 1 (1=slow, 8=very fast) to prevent cell detachment.*
3. Add 80 µl of assay medium to the rest of the wells.

   Δ CRITICAL *Set the dispensing speed at 1 (1=slow, 8=very fast) to prevent cell detachment.*

4. Label **titration plate** with virus name and put it back at 37°C until the end of virus incubation period.

B) Dilution series of YFV/mCherry virus stock.

Timing 10 min followed by 1h of incubation (day 0)

Δ CRITICAL *Hold the tips perpendicular to plate and pipette at the bottom of wells during dispensing and mixing.*

1. Mix virus stock by pipetting up and down and transfer 60 µl of virus to column 2 (wells: B-G) in a 96-well round bottom plate.
2. Fill all the remaining wells with 40 µl of assay medium (Table 2).

Table 2. Liquid volumes required for dilution series in virus plate.

Row	Column 2, virus stock in well (µl)	Columns 3-11, assay medium in well (µl)
A		40
B	60	40
C	60	40
D	60	40
E	60	40
F	60	40
G	60	40
H		40

3. Mix virus in column 2 using a P100 electronic 8-channel micropipette and discard the tips.
4. Transfer 20 µl from column 2 to column 3 using a new set of tips, repeat mixing and discard the tips.
5. Dilute virus further as indicated in step 3.2: B4 up to column 11 (Figure 2). Discard the final 20 µl.
6. Gently tap the sides of the plate several times to allow mixing.
7. Label this plate **virus plate** and incubate for 60 min at 37°C and 5% CO_2.

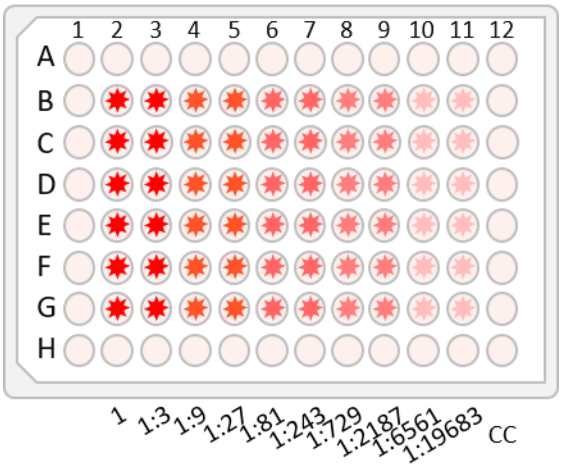

Figure 2. Layout of a virus plate (round bottom plate). Dilution factors of the virus (red octagons) are mentioned beneath the plate. CC is a cell control.

C) Inoculation of cells with serially diluted virus.

Timing 5 min (day 0)

1. At the end of the incubation period, take the virus and titration plates out of the incubator.
2. Gently mix virus dilutions in row G (mode=P/M, aspiration speed=3, dispensing speed=3, mixing volume=20 µl, times=2), transfer 20 µl of it to the cells, and repeat mixing.

   Δ CRITICAL *Hold the tips perpendicular to the plate while mixing in a round-bottom plate.*

   Δ CRITICAL *Hold the tips at a 45 degree angle against the well wall and slightly immersed in medium while transferring the mix to cells.*
3. Repeat step 3.2: C2 for rows F and E with the same set of tips.
4. Proceed to rows D, C and A as in step 3.2: C2-C3 with a new set of tips (Figure 3).
5. Incubate titration plate at 37°C and 5% CO_2 for 3 days.

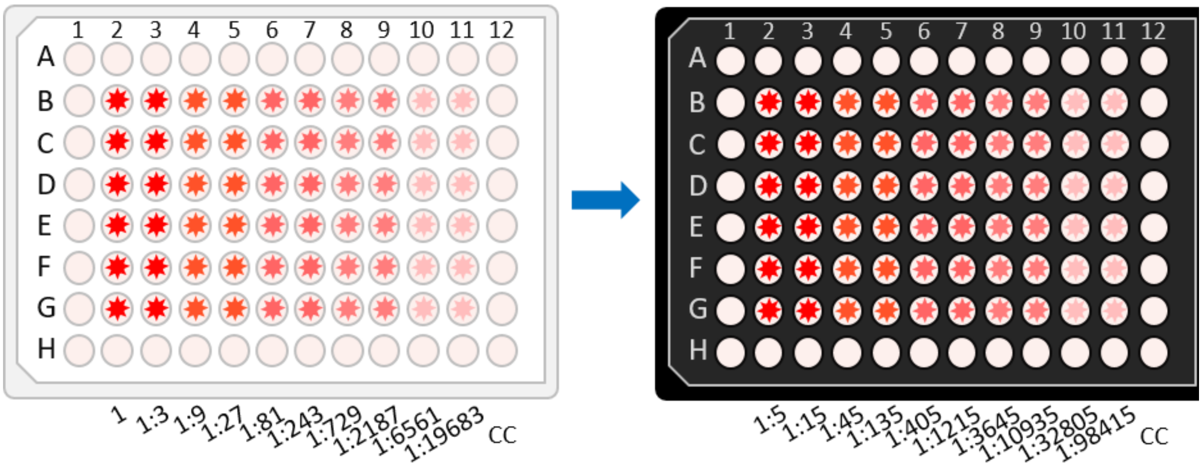

Figure 3. Transfer pre-incubated viral serial dilutions to the cells (titration plate; black, clear bottom).

Dilution factors of the virus (red octagons) are indicated beneath the plates. CC is a cell control.

3.3. Day 3 post-infection: cell fixation.

Timing 30 min followed by 30 min of drying (day 3)

1. Add 100 µl of fixation solution to each well in the titration plate.

   ! CAUTION Fixation should be preferably performed in a fume hood to minimize inhalation of vapor formaldehyde.

2. Fix for 15 min at RT in the dark.
3. Aspirate fixation solution using the 8-channel vacuum manifold.
4. Wash cells with 200 µl of DPBS and decant the liquid.
5. Invert the plate upside-down and gently tap it onto a tissue paper to remove remaining liquid droplets.
6. Let the plate dry for at least 30 min in the dark at RT until imaging.

3.4. Quantify fluorescence with Immunospot reader.

Timing 30 min (day 3)

1. Switch the optics selection rod to fluorescence mode and start the fluorescence-X software suite.
2. Check the instrument settings as indicated in Table 5.

Table 5. Settings for Immunospot software to scan spots formed by YFV/mCherry.

Plate form factor	96 Well Greiner black no lid no liquid
Filter ID	Filter 600
Focus	4000
Zoom	1.7086x
Excitation band selector (nm)	570
Fixed Exposure	No autoexposure (checked)
Gain	25
Time	4000 µs

2. Insert the dried plate without lid and start scanning.

   ! CAUTION *Plates with fixed cells may have traces of formaldehyde which is harmful to humans. Wear gloves, and avoid contact with eyes and skin while handling the plates.*

3. When scanning is complete, switch to the BioSpot software suite and select “Basic Count” option.

4. Load the scanned plate image and counting parameters for YFV/mCherry as shown in Table 6.

Table 6. Counting parameters to estimate YFV/mCherry-formed fluorescent spots in the Biospot basic counting mode.

Sensitivity	229
Minimal spot size	0.0004 mm^2
Maximal spot size	12.0370 mm^2
Oversized Spots	Estimated
Spot Separation	1.00
Diffuseness	Large
Overdeveloped area handling	Active
Objects	Inverted, diffuse spots, high background
Background Balance	40
Weight Function Shape	0.5
Edge Compensation Level	1.0

5. Run the counting algorithm. A representative image of a counted plate is given in Figure 10.

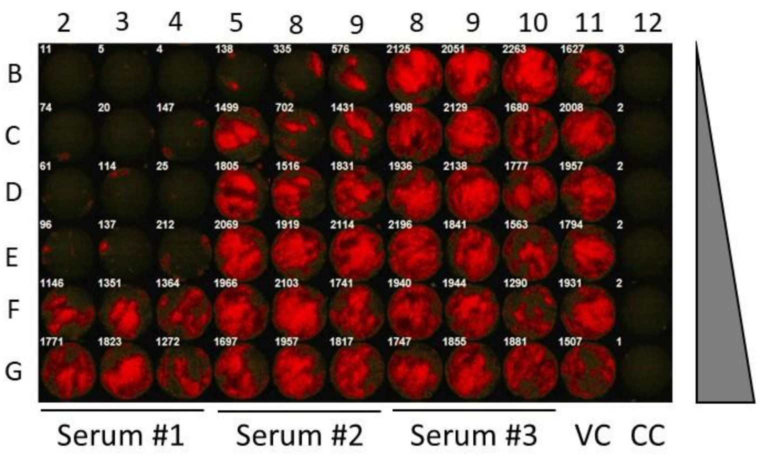

Figure 10. Typical SNT^{FLUO} assay plate read-out at day 3 post-infection. VC and CC represent virus and cell controls, respectively.

3.5. Determination of optimal titer for infection.

Timing 1 min (day 3 or later)

1. Determine Z'-factor.
2. Use the following quality requirements:

   Saturation point = 2000

   Minimum Z'-factor = 0.50
3. Check which dilutions produce sufficient quality. The highest virus dilution with sufficient quality should be the optimal titer for the assay. Use this virus dilution in SNT^{FLUO}.

○ **IMPORTANT:** MOI of the selected virus dilution will correspond to ~0.02 as determined by classical plaque assay.

4. YFV/mCherry serum neutralization assay (SNT^{FLUO}) (Figure 5).

Timing 5 days

Δ CRITICAL:

- *All the steps must be performed in a class II biosafety cabinet, using proper aseptic technique.*
- *Allow assay medium to equilibrate to RT prior to use.*

- *Allow serum samples and YFV/mCherry virus stock to thawed at RT for at least 15 min.*
- *Aspiration is performed with the 8-channel vacuum manifold and the vacuum pump is set to low suction power (2 LED's, 250 mbar underpressure). Hold the tips at an angle of 45 degrees and slowly move down against the well wall until the bottom is reached.*
- *Dispensing steps are performed with 12-channel electronic micropipettes (P100, P1250). Hold the tips at a 45 degree angle against the well wall during dispensing and set the dispensing speed at 1 (1=slow, 8=very fast), unless otherwise mentioned.*

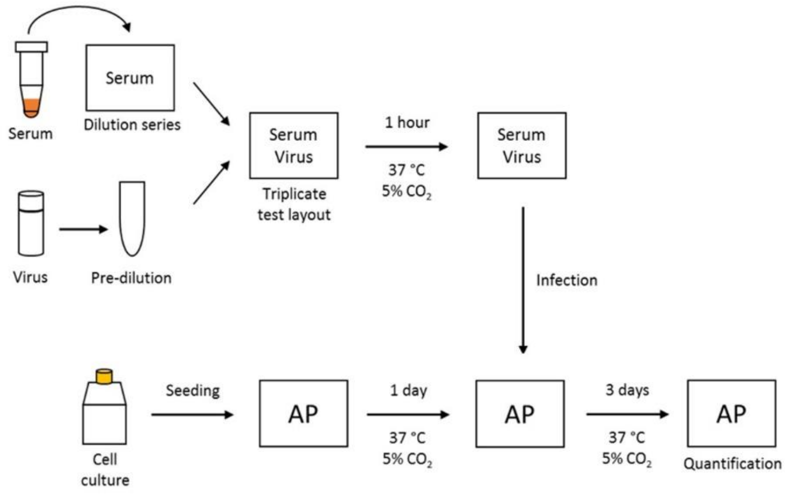

Figure 5. Schematic overview of the complete SNT^{FLUO} procedure. Serum is pre-incubated with virus before transferring the mix to BHK-21J cells, and fluorescent cells are quantified 3 days post infection using a Immunospot reader. Serum samples with neutralizing antibodies will have less fluorescent spots compared to the virus control. AP is an assay plate.

4.1. Preparation of sera and cell plates.

Timing 50 min – 2h (day -1)

A) Preparation of serum samples.

Timing 30 min followed by 30 min of heat-inactivation (day -1)

1. Fill the barcode number, the layout of plate and serum samples name in an excel sheet.
2. Mix first serum sample by pipetting up and down 6-times.

3. Transfer 15 µl of serum to the first well of a sterile 48-well plate (A1).

4. Mix the second serum sample and transfer 15 µl to the next well (A2).

5. Proceed with the other samples in a similar manner and fill the 48-well plate as shown in Figure 6.

6. Seal each column with a strip of 8-caps and attach a unique barcode on each plate.

7. Place the plate in the thermocycler and incubate sera for 30 minutes at 56°C.

   Δ CRITICAL *Heating serum is required for complement inactivation.*

8. After incubation, quickly spin the plate during 30 sec at 400 xg to remove condensates.

9. Label 48-well plate with a unique barcode.

   □ **PAUSE POINT** *Heat-inactivated serum aliquots can be stored at -20°C for several months. This allows simultaneous testing of samples collected at different time points to ensure read-out consistency. All collected samples should be subjected to the same number of freeze–thaw cycles (preferably no more than two) to ensure consistent reporter readings.*

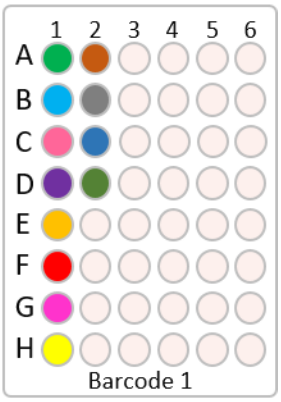

Figure 6. Layout of a 48-well serum plates. Each colour represents a unique serum sample. Up to 48 unique samples can be stored in a single 48-well plate.

B) Cell seeding.

Timing 15 min – 1h (day -1)

1. Seed 1 x 10^4 cells/well in 96-well black plates in assay medium as given in 3.1. Note: one cell plate is sufficient to analyze 3 serum samples in triplicate.

2. Incubate the plates overnight at 37°C and 5% CO_2 to adhere cells to the bottom.

4.2. Serum neutralization.

Timing 30 min – 3h (day 0)

A) Medium replacement.

Timing 5 – 30 min (day 0)

6. Replace medium as explained in 3.2: A.
7. Label assay plates **(assay plate 1, 2, etc)** and put them back at 37°C until the end of serum incubation period.

B) Dilution series of serum samples.

Timing 10 – 30 min (day 0)

Δ CRITICAL *Hold the tips perpendicular to the plate and pipette at the bottom of wells during dispensing and mixing.*

1. Fill a 96-well round bottom plate with assay medium according to Table 3.
2. Mix heat-inactivated serum samples by pipetting up and down.
3. Transfer 7 µl of serum samples to row B as shown in Figure 7A and repeat mixing. Note: up to 12 samples can be diluted in one plate.

Table 3. Liquid volumes required for dilution series in serum plate.

Row	Dilution factor	Assay medium in well (µl)	Serum (µl)
A		70	
B	15	98	+7
C	45	70	35
D	135	70	35
E	405	70	35
F	1215	70	35
G	3645	70	35
H		70	

4. Mix serum in row B (increase mixing volume to 35 µl) and discard tips.
5. Transfer 35 µl from row B to row C using a micropipette with a new set of tips and repeat mixing.
6. Dilute serum samples further up to row G (Table 3, Figure 7B) and discard the final 35 µl.
7. Label this plate **(serum plate 1, 2, etc)**.

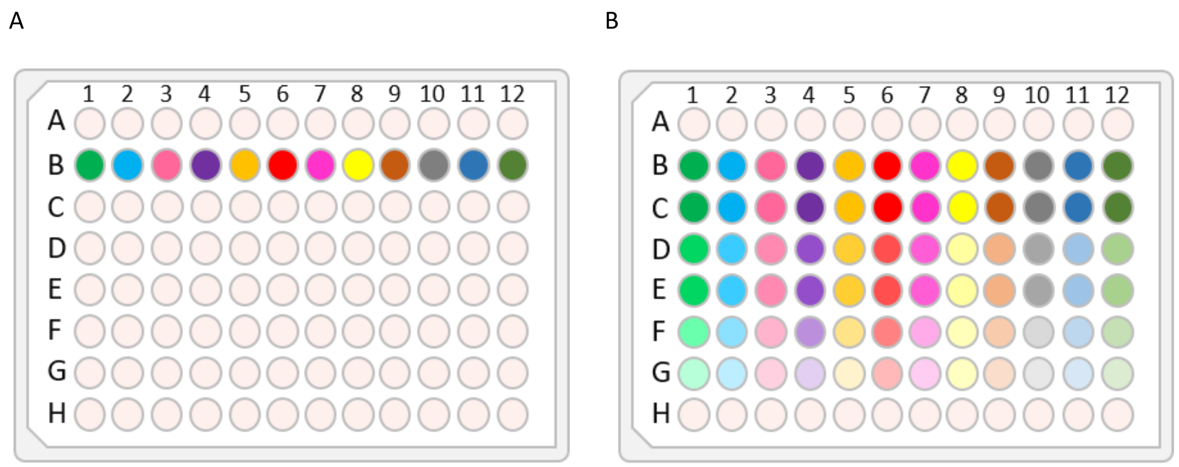

Figure 7. Layout of serum plate after addition of 12 different serum samples in row B (A) and after performing 3-fold serial dilutions (B). Unique serum samples and their dilutions are represented with different colours and their shades respectively.

C) Virus pre-dilution.

Timing 5 min (day 0)

1. Prepare virus pre-dilution in a Falcon tube. Note: 2 ml of virus suspension is required per plate.

Δ CRITICAL *Virus will be diluted 1:2 with serum and later 1:5 on cells, therefore preparing 10-fold concentrated virus pre-dilution is important (e.g. the final titer for YFV/mCherry virus stock is 1:1200, so the pre-dilution must be 1:120).*

2. Mix virus pre-dilution by inverting the Falcon tube several times and decant it into a reagent reservoir.

D) Co-incubation of serum dilutions (4.2: step B) with virus pre-dilution (4.2: step C).

Timing 5 min – 1h (day 0)

1. Take a new 96-well round bottom plate and label it **(serum/virus plate 1)**. Note: up to 3 sera in 3 replicates can be tested on a single serum/virus plate.
2. Mix serial dilutions of the first test-serum (mode=P/M, aspiration speed=8, dispensing speed=8, mixing volume=40 µl, times=2) and transfer 3-times 18 µl from the **serum plate** to the **serum/virus plate** as depicted in Figure 8A.

Δ CRITICAL *Use a new set of filter tips for each serum and hold them in the bottom of the wells during dispensing.*

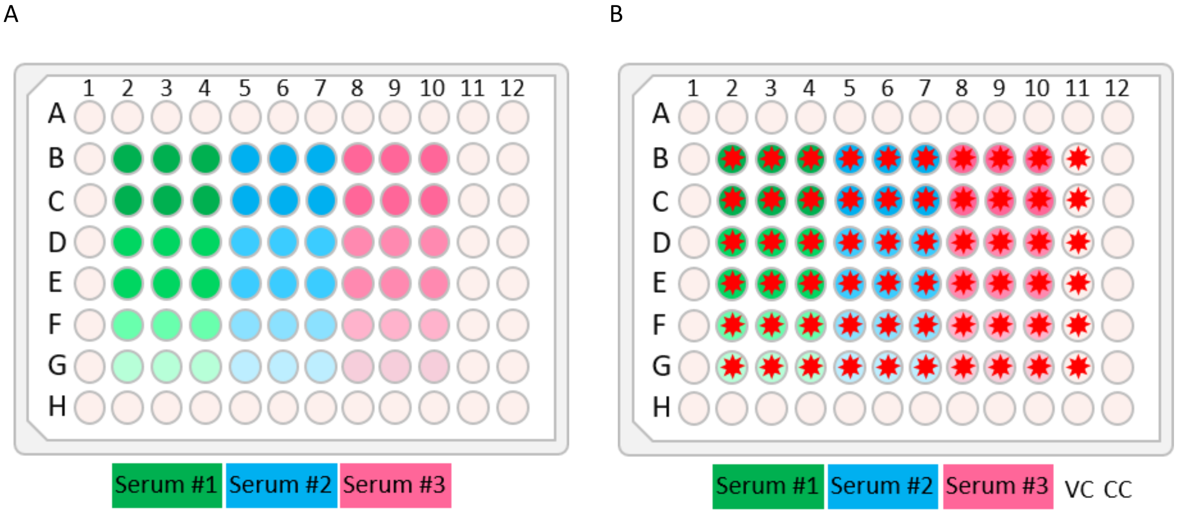

Figure 8. Layout of serum/virus plate after addition of 3 serum samples in triplicate (A) and after inoculation with virus (B). VC and CC indicate virus and cell controls respectively.

3. Repeat step 4.2: D2 for the next two serially diluted sera.
4. Add 18 µl of assay medium to VC wells (column 11).

   Δ CRITICAL *Hold the tips in the middle of the wells during dispensing.*
5. Add 36 µl of assay medium to CC wells (column 12).
6. Add 18 µl of pre-diluted virus to all wells except CC. Start from row G and work towards row B *(Figure 8B)*.

   Δ CRITICAL *Hold the tips at a 45 degree angle against the well wall during dispensing to avoid cross-contamination with sera.*
7. Gently tap the sides of the plate several times to allow mixing.
8. Place serum/virus plate #1 at 37°C and 5% CO_2 for 60 minutes.
9. During the incubation time, prepare serum/virus plate #2 by following steps 4.2: D1-D8. Repeat these steps until all the serum/virus plates are ready.

   Δ CRITICAL *Leave at least 3 min gap between each serum/virus plate to ensure equal incubation time prior to proceeding with step 4.2: E.*

E) Inoculation of cells with virus-serum complexes.

Timing 5 min – 1h (day 0)

1. At the end of the incubation period, take the first serum/virus and assay plates out of the incubator.

   Δ CRITICAL *Work plate by plate and take only one of each plate out of incubator at a time.*

2. Gently mix serum/virus complex in row G (mode=P/M, aspiration speed=3, dispensing speed=3, mixing volume=20 µl, times=2), transfer 20 µl to the cells in row G (**assay plate 1**), and repeat the mixing.
3. Repeat step 4.2: E2 for rows F and E with the same set of tips and discard them.
4. Proceed to rows D, C and A as in step 4.2: E2 with a new set of tips (Figure 9).
5. Place the assay plate 1 in incubator.
6. Repeat steps 4.2: E1-E5 for the other plates.

   Δ CRITICAL *Begin with the second serum/virus and assay plates when the respective incubation time of 60 minutes is over.*

7. Incubate the assay plates at 37°C and 5% CO_2 for 3 days.

Table 4. Dilution factors of serum after each step. Initial dilution in assay medium (4.2: B), after addition of virus (4.2: D) and final dilution on cells (4.2: E).

Row	In medium	With virus	On cells
B	15	30	150
C	45	90	450
D	135	270	1350
E	405	810	4050
F	1215	2430	12150
G	3645	7290	36450

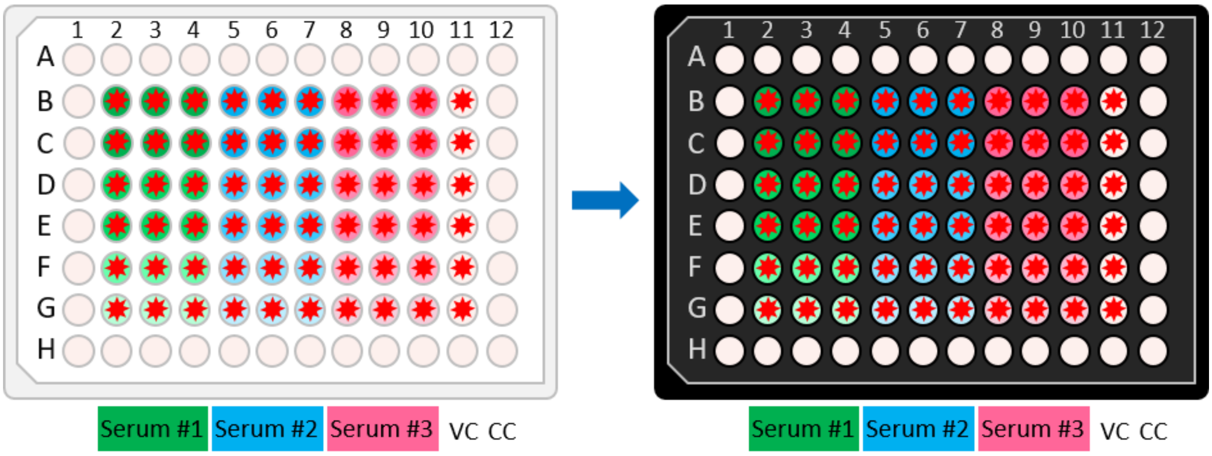

Figure 9. Transfer pre-incubated serum/virus mix to the cells (assay plate; black, clear bottom). VC and CC indicate virus and cell controls respectively.

4.3. Cell fixation.

Timing 1h 30 min followed by 30 min of drying (day 3)

Proceed as in Section 3.3.

4.4. Quantification of fluorescent spots using Immunospot reader.

Timing 2h (day 3)

Proceed as in Section 3.4.

4.5. Analyse data using Genedata Screener.

Timing 1h (day 3 or later)

Δ CRITICAL *Genedata Screener calculates the Z' factor (a measure for plate quality) for each plate from the VC and CC wells. Plates with Z' factor ≥ 0.5 are of sufficient quality for reliable activity calculation.*

Δ CRITICAL *When some of the plates have Z' factor between 0.1-0.5, they require additional evaluation under fluorescence microscope to determine the integrity of cell monolayer and/or possible debris. If cell monolayer is intact and/or wells with debris are excluded from analysis, the data can be trusted. Otherwise repeat the assay starting from Section 4.*

Δ CRITICAL *If Z' factor of most of the plates is <0,5, this indicates that the titer of the virus stock declined and needs to be reassessed again. Proceed with Section 3 and use the newly determined virus titer for the following SNT^{FLUO} assays.*

Timing

Procedure 1: Cell culture maintenance: 15-30 min (once a week)

Procedure 2: Production of YFV17D/mCherry virus stock: 5-7 days

Cell seeding, section 2.1: 15 min (day -1)

Transfection, section 2.2: 30 min (day 0)

Medium replacement, section 2.3: 5 min (day 1)

Harvesting, section 2.4: 1h (day 3-5)

Procedure 3: Titration of YFV/mCherry virus stock: 5 days

Cell seeding, section 3.1: 15 min (day -1)

Virus titration, section 3.2: 1h 20 min (day 0)

A) Medium replacement: 5 min (day 0)

B) Dilution series of YFV/mCherry virus stock: 10 min followed by 1h incubation (day 0)

C) Inoculation of cells with serially diluted virus: 5 min (day 0)

Cell fixation, section 3.3: 30 min followed by 30 min drying (day 3)

Quantify fluorescence with Immunospot reader, section 3.4: 30 min (day 3)

Determination of optimal titer for infection, section 3.5: 1 min (day 3 or later)

Procedure 4: YFV/mCherry serum neutralization assay (SNT^{FLUO}): 5 days

Preparation of sera and cell plates, section 4.1: 50 min – 2h (day -1)

A) Preparation of serum samples: 5 – 30 min followed by 30 min heat-inactivation (day -1)

B) Cell seeding: 15 min – 1h (day -1)

Serum neutralization, section 4.2: 30 – 3h (day 0)

A) Medium replacement: 5 – 30 min (day 0)

B) Dilution series of serum samples: 10 – 30 min (day 0)

C) Virus pre-dilution: 5 min (day 0)

D) Co-incubation of serum dilutions (4.2: step B) with virus pre-dilution (4.2: step C): 5 min – 1h (day 0)

E) Inoculation of cells with virus-serum complexes: 5 min – 1h (day 0)

Cell fixation, section 4.3: 1h 30 min followed by 30 min drying (day 3)

Quantification of fluorescent spots using Immunospot reader, section 4.4: 2h (day 3)

Data analysis using Genedata Screener, section 4.5: 1h (day 3 or later)
